# GIP-Overexpressing Mice Demonstrate Reduced Diet-Induced Obesity and Steatosis, and Improved Glucose Homeostasis

**DOI:** 10.1371/journal.pone.0040156

**Published:** 2012-07-03

**Authors:** Su-Jin Kim, Cuilan Nian, Subashini Karunakaran, Susanne M. Clee, Carlos M. Isales, Christopher H. S. McIntosh

**Affiliations:** 1 Department of Cellular & Physiological Sciences and the Diabetes Research Group, Life Sciences Institute, University of British Columbia, Vancouver, British Columbia, Canada; 2 Departments of Orthopaedic Surgery and Cellular Biology and Anatomy, Georgia Health Sciences University, Augusta, Georgia, United States of America; University of Bremen, Germany

## Abstract

Glucose-dependent insulinotropic polypeptide (GIP) is a gastrointestinal hormone that potentiates glucose-stimulated insulin secretion during a meal. Since GIP has also been shown to exert β-cell prosurvival and adipocyte lipogenic effects in rodents, both GIP receptor agonists and antagonists have been considered as potential therapeutics in type 2 diabetes (T2DM). In the present study, we tested the hypothesis that chronically elevating GIP levels in a transgenic (Tg) mouse model would increase adipose tissue expansion and exert beneficial effects on glucose homeostasis. In contrast, although GIP Tg mice demonstrated enhanced β-cell function, resulting in improved glucose tolerance and insulin sensitivity, they exhibited reduced diet-induced obesity. Adipose tissue macrophage infiltration and hepatic steatosis were both greatly reduced, and a number of genes involved in lipid metabolism/inflammatory signaling pathways were found to be down-regulated. Reduced adiposity in GIP Tg mice was associated with decreased energy intake, involving overexpression of hypothalamic GIP. Together, these studies suggest that, in the context of over-nutrition, transgenic GIP overexpression has the potential to improve hepatic and adipocyte function as well as glucose homeostasis.

## Introduction

Until recently, the major drugs utilized for treating type 2 diabetes (T2DM) were members of the sulfonylurea or meglitinide family, targeted at stimulating insulin secretion, biguanides (*e.g.* metformin) or thiazolidinediones, for reducing hepatic glucose output and insulin resistance, and α-glucosidase inhibitors for lowering carbohydrate digestion [1]–[2]. However, since sub-optimal glucose control or monotherapy failure often occur, combination therapy or, ultimately, insulin treatments are often needed [3]–[4]. Additional approaches have therefore been sought, including drugs based on the actions of the two incretin hormones, Glucose-dependent Insulinotropic Polypeptide (GIP) and Glucagon-like Peptide-1 (GLP-1), that convey insulinotropic signals from the gut to the pancreatic islets during a meal. Both GIP and GLP-1 have been shown to exert a number of additional anti-diabetic actions, including promotion of β-cell survival and proliferation, and GLP-1 also inhibits glucagon secretion and reduces both gastric emptying and food intake [5]–[9]. Long-acting GLP-1 analogs (mimetics) and highly selective inhibitors of the incretin-degrading enzyme, dipeptidyl peptidase-4 (DPP-IV), are now widely used as T2DM therapeutics.

**Figure 1:**
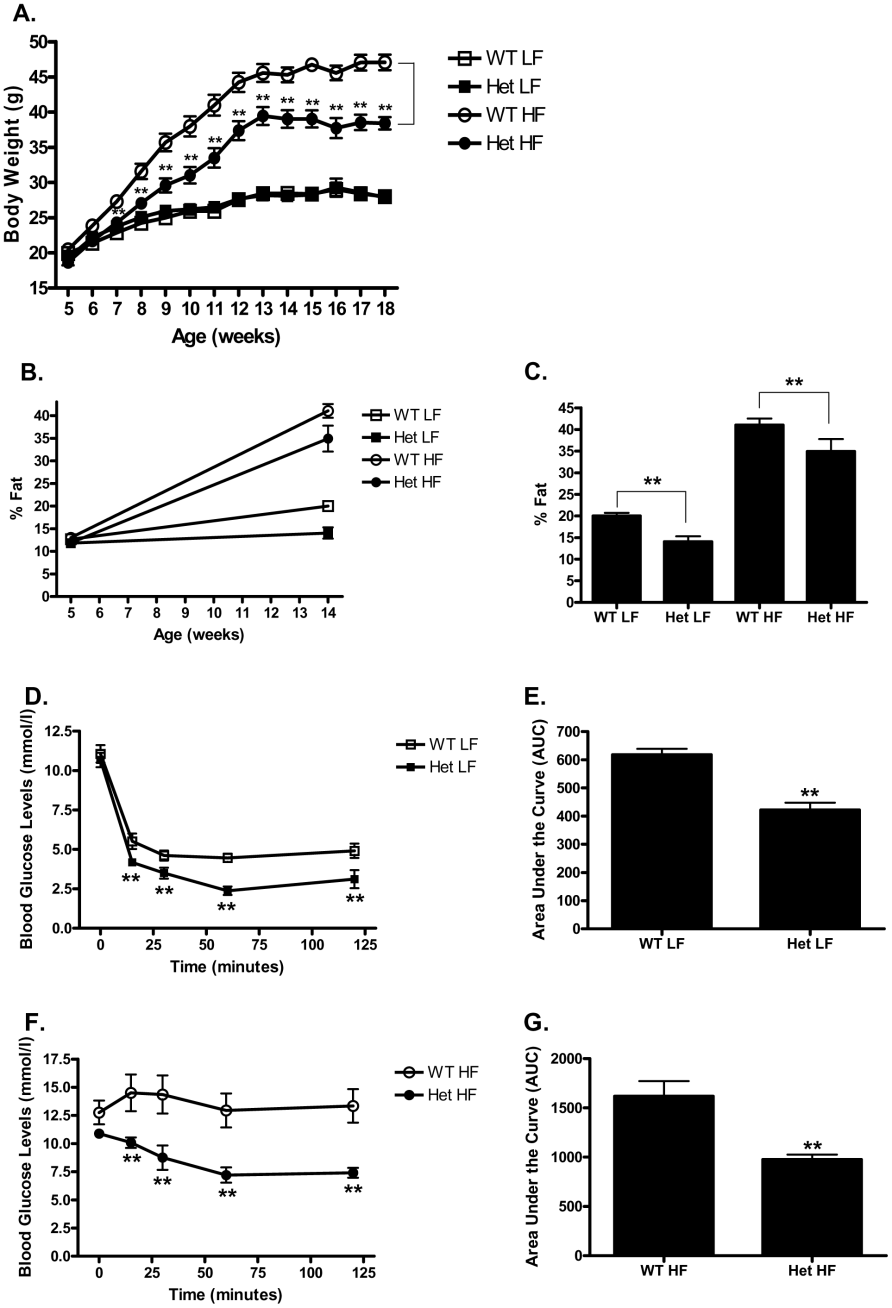
Het GIP Tg mice exhibit reduced weight gain and insulin resistance on HF diet. Het GIP Tg (Tg/+) and non-transgenic WT littermates (+/+, 5 weeks old) were placed on either a low fat (LF) or high fat (HF) diet, and 25 mM ZnSO_4_ was added to the drinking water of both Het GIP Tg and WT mice (*n* = 6/group). **A.** Body weight gain in Het GIP Tg and WT mice placed on LF or HF diet. **B.** Percentage fat gain in WT and Het GIP Tg mice. **C.** Percentage fat of WT and Het GIP Tg mice on 14 weeks old. **D and F.** Glucose levels during an insulin tolerance test in LF (**D**)- and HF (**F**) diet-fed Het GIP Tg and WT littermates. After 8 weeks of feeding, WT and Het GIP Tg mice (13 weeks old, *n* = 6/group) were fasted for 4 hours and subsequently injected with 0.75 U/kg insulin. Blood glucose levels measured at 0, 15, 30, 60 and 120 min following insulin administration. **E and G.** Quantification of AUC for the total glycemic excursions in **D** and **F.** All data represent the mean ± S.E.M. and significance was tested using ANOVA with a Newman-Keuls post hoc test or Student’s *t* test, where ** represents p<0.05 *vs* indicated group or WT littermates.

The majority of studies on GIP to date have focused on its insulinotropic actions and β-cell functions, and long-term administration of DPP-IV resistant GIP analogues has been shown to markedly improve glucose tolerance in normal and diabetic rats, as well as in high fat (HF) fed mice [10]–[12]. Additionally, subcutaneous administration of D-Ala^2^-GIP_1–30_ to Zucker diabetic fatty rats resulted in potent anti-diabetic effects that included reduced β-cell apoptosis and improved β-cell function and glycemic control [13]. There is also strong evidence supporting a role for GIP in the regulation of fat metabolism that is consistent with its anabolic characteristics [9]. Among the adipocyte actions of GIP that have been identified are stimulation of adipogenesis, enhancement of lipoprotein lipase (LPL) activity, and increased lipolysis and fatty acid reesterification [14]–[21]. Studies showing that homozygous GIP receptor knockout (KO) mice (GIPRKO) were resistant to obesity when fed a HF diet [22] and the presence of K-cell hyperplasia and elevated GIP and insulin levels in HF fed rodents, led to the suggestion that GIP may contribute to the development of obesity, with associated insulin resistance and glucose intolerance [23]–[24]. Consequently, it has been proposed that GIP antagonist treatment [25], reducing circulating GIP levels with K-cell ablation [26] or vaccination against GIP [27] may be beneficial treatments for obesity. However, the question as to whether long-term elevation of GIP production causes detrimental pro-obesity effects has not been directly addressed and we have therefore examined responses of transgenic overexpression of GIP (GIP Tg) mice to HF diet feeding. The results demonstrate that, in contrast to expectations, transgenic overexpression of GIP had major beneficial effects on both glucose and fat metabolism.

**Figure 2:**
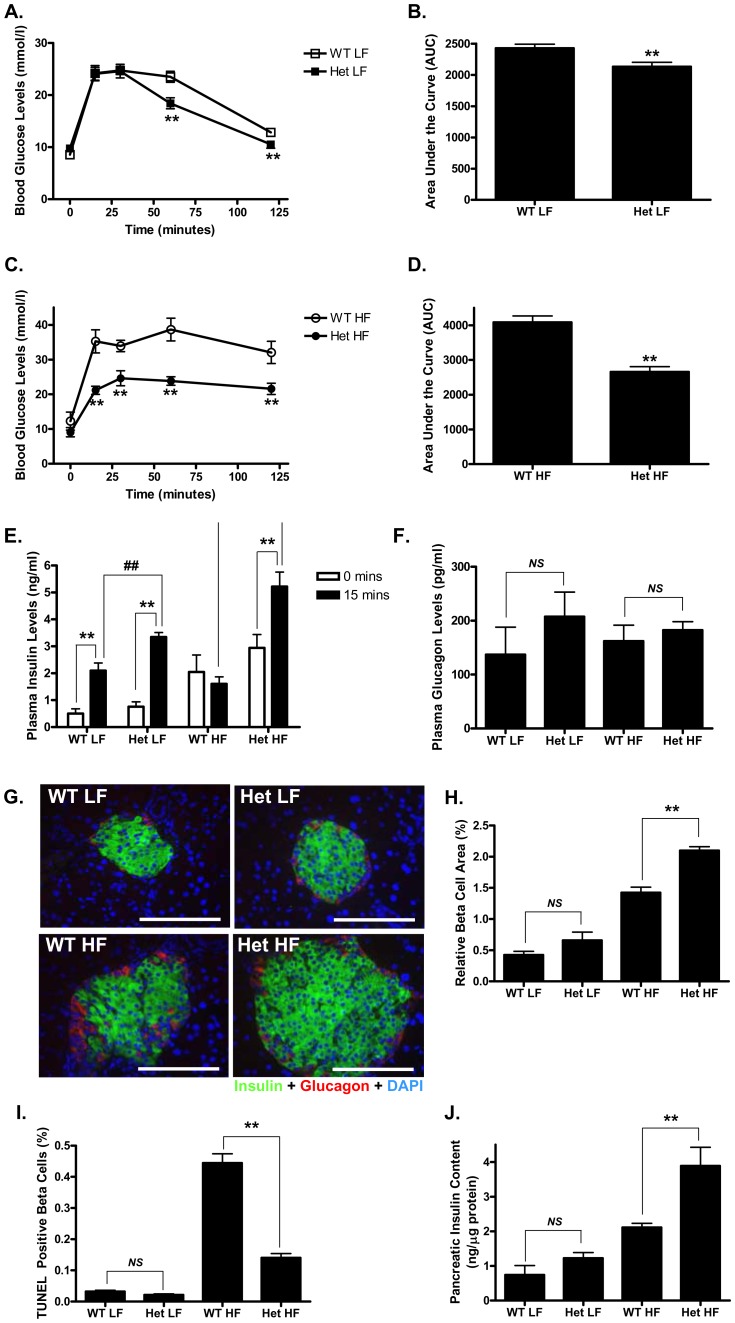
Het GIP Tg mice were resistant to HF diet-induced glucose intolerance. A and C. Glucose levels during a glucose tolerance test in LF (**A**)- and HF (**C**) diet-fed Het GIP Tg and WT littermates. After 10 weeks of LF and HF feeding, WT and Het GIP Tg mice (15 weeks old, *n* = 6/group) were fasted for 4 hours and blood glucose levels measured at 0, 15, 30, 60 and 120 min following a glucose challenge (2 g/kg). **B and D.** Quantification of AUC for the total glycemic excursions in **A** and **C**. **E–F.** Plasma insulin (**E**) and glucagon (**F**) during a glucose tolerance test. **G.** Representative pancreatic images from WT and Het GIP Tg mice (18 weeks old) following 13 weeks of LF and HF feedings. Pancreatic sections were stained with insulin (green), glucagon (red) and DAPI (blue). The scale bar indicates 100 µm. **H.** Relative β-cell area expressed as the percentage of sectional pancreas area as described in *Experimental Procedures*. **I.** The percentage of β-cells undergoing apoptosis as determined by TUNEL positive nuclei. **J.** Total pancreatic insulin content in WT and Het GIP Tg mice (18 weeks old) following 13 weeks of LF and HF feedings. All data represent the mean ± S.E.M. and significance was tested using ANOVA with a Newman-Keuls post hoc test or Student’s *t* test, where ** and ## represents p<0.05 *vs* indicated group or WT littermates; *NS* represents not significant.

## Results

### 1. Mice Overexpressing GIP Exhibited Reduced HF Diet-induced Weight Gain and Adiposity

To determine the effect of GIP overexpression on fat development, heterozygous (Het) GIP Tg (Tg/+) and WT littermates (+/+) were placed on low fat (LF) or high fat (HF) diets from 5 to 18 weeks of age. For induction of GIP expression, 25 mM ZnSO_4_ was added to the drinking water. As expected, WT mice fed a HF diet demonstrated a markedly greater increase in body weight, compared to those on a LF diet, over the course of the study. Although Het GIP Tg mice on a HF diet also gained more weight than those on a LF diet, from 7 weeks on the weight gain was significantly less than HF fed WT mice, as was the final weight at 18 weeks (38.4±0.9 g *vs* 47.1±1.1 g) (Figure 1A). Neither body length nor lean body mass differed between the Het GIP Tg mice and WT littermates (Data not shown), however, percent fat mass was significantly decreased compared to WT mice in 14 week old Het GIP Tg mice on both LF and HF diets (Figures 1B and 1C).

**Figure 3:**
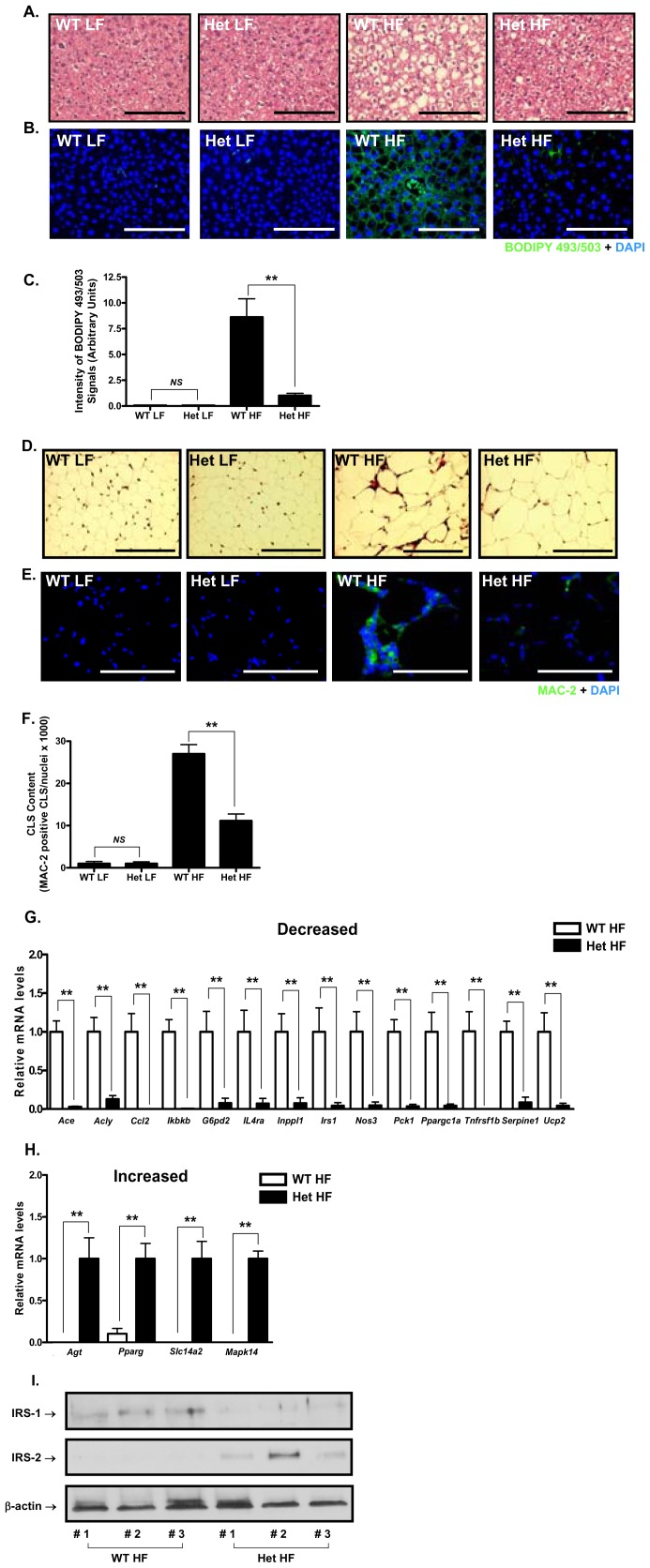
GIP overexpression alters hepatic and adipose tissue responses to a high fat diet. **A-F.** Histological analyses of liver (**A, B and C**) and epididymal white adipose tissue (eWAT, **D, E and F**) from Het GIP Tg and WT littermates (18 weeks old) following 13 weeks of LF and HF feedings. Liver sections were stained with H&E (**A,** scale bar = 100 µm) and the lipid body stain BODIPY 493/503 (**B,** scale bar = 100 µm). **C.** BODIPY 493/503 positive signals. BODIPY 493/503 positive signals were quantified as described in *Experimental Procedures*. Fat sections were stained with H&E (**D,** scale bar = 100 µm) and antibodies against the macrophage antigen MAC-2 (**E,** scale bar = 25 µm). **F.** eWAT Crown-Like Structure (CLS) content. MAC-2 positive CLS content was quantified as described in *Experimental Procedures*. **G and H.** Gene expression profiles. Total RNA was extracted from eWATs of Het GIP Tg and WT littermates on HF feeding, and RT^2^ Profiler PCR Arrays were performed to simultaneously quantify mRNA expression levels of multiple genes. **I. Altered Insulin-Receptor Substrate 1 & 2 protein expression.** Proteins were extracted from eWATs of Het GIP Tg and WT littermates on HF feeding, and Western blot analyses performed using antibodies against IRS-1, IRS-2, and β-actin. All data represent the mean ± S.E.M. and significance was tested using ANOVA with a Newman-Keuls post hoc test or Student’s *t* test, where ** represents p<0.05 *vs* indicated group; *NS* represents not significant.

### 2. Improved Insulin Sensitivity, Glucose Tolerance and β-cell Function in Het GIP Tg Mice

To evaluate the effect of GIP overexpression on insulin sensitivity, intraperitoneal insulin tolerance tests (IPITTs) were performed following 8 weeks of LF/HF feeding (*n* = 6 animals/group). Fasting glucose in Het GIP TG mice on either LF or HF diets did not differ from WT mice. However, although the Het GIP Tg mice became markedly insulin resistant on the HF diet, they showed significantly enhanced insulin sensitivity compared to their WT littermates on both LF and HF diets (Figures 1D–1G).

Intraperitoneal glucose tolerance tests (IPGTTs) were performed following 10 weeks of LF/HF feeding (*n* = 6 animals/group). As shown in Figures 2A–2D, Het GIP Tg mice showed improved glucose tolerance compared to WT littermates on both of LF and HF diets. Het GIP Tg mice on LF diet showed significantly enhanced insulin secretory responses to glucose stimulation, compared with WT littermates. On the HF diet, the WT littermates almost lost their insulin secretory responses to glucose, whereas Het GIP Tg mice on the same diet showed robust insulin responses following the glucose challenge (Figure 2E). Glucagon levels did not differ between Het GIP Tg and WT littermates (Figure 2F). Subsequent studies, on islets collected at the termination of the experiment, demonstrated that β-cell area and total pancreatic insulin content were significantly increased in HF fed Het GIP Tg mice, compared with WT littermates (Figures 2H and 2J) and this was accompanied by significantly decreased β-cell apoptosis (Figure 2I). Mean β-cell area and pancreatic insulin content were increased in Het GIP Tg mice on LF feeding, although differences did not reach statistical significance (Figures 2G and 2H). Together these results strongly suggest that overexpression of GIP in mice exerts beneficial effects on the regulation of insulin sensitivity and glucose tolerance, and the improvement was at least partially due to β-cell compensation resulting from enhanced β-cell function.

**Figure 4:**
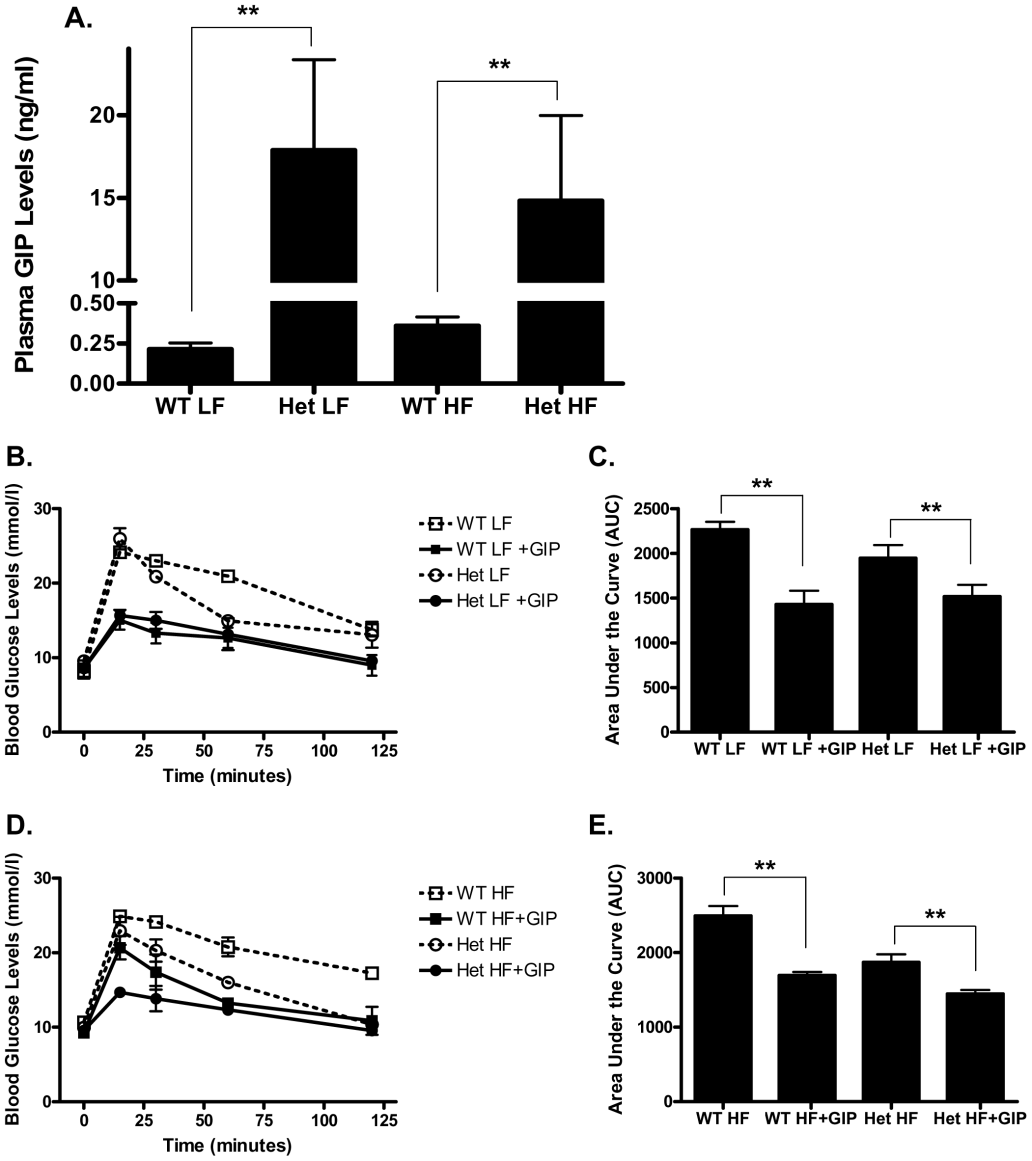
Het GIP Tg mice retain responsiveness to GIP. A. Plasma GIP levels. After 13 weeks of LF or HF diet feeding, circulating GIP levels were determined in Het GIP Tg and WT littermates (*n* = 5∼6/group). **B and D.** Effects of GIP on glycemic excursions in LF (**B**)- and HF (**D**) diet-fed Het GIP Tg and WT littermates. After 13 weeks of feeding, WT and Het GIP Tg mice (*n* = 5∼6/group) were fasted for 4 hours, and PBS or GIP (24 nmol/kg) were administered immediately prior to glucose loading (2 g/kg). Blood glucose levels were measured at 0, 15, 30, 60 and 120 min following the glucose challenge. **C and E.** Quantification of AUC for the total glycemic excursions in **B** and **D.** All data represent the mean ± S.E.M. and significance was tested using ANOVA with a Newman-Keuls post hoc test, where ** represents p<0.05 *vs* WT littermates or ndicated group.

### 3. GIP Overexpression Altered Hepatic and Adipose Tissue Responses to a High Fat Diet

Liver sections from WT mice at 18 weeks exhibited marked steatosis following the HF diet, but the GIP overexpressing mice showed a remarkable attenuation of hepatic lipid accumulation (Figures 3A–3C). As expected, adipocytes in epididymal white adipose tissue (eWAT) from both WT and Het GIP Tg mice exhibited greatly increased size following the HF diet (Figure 3D). Adipose tissue macrophage infiltration was also greatly increased in WT mice following 13 weeks of HF feeding, but tissue from Het GIP Tg mice showed substantially less infiltration of macrophages (Figures 3E–3F).

**Figure 5:**
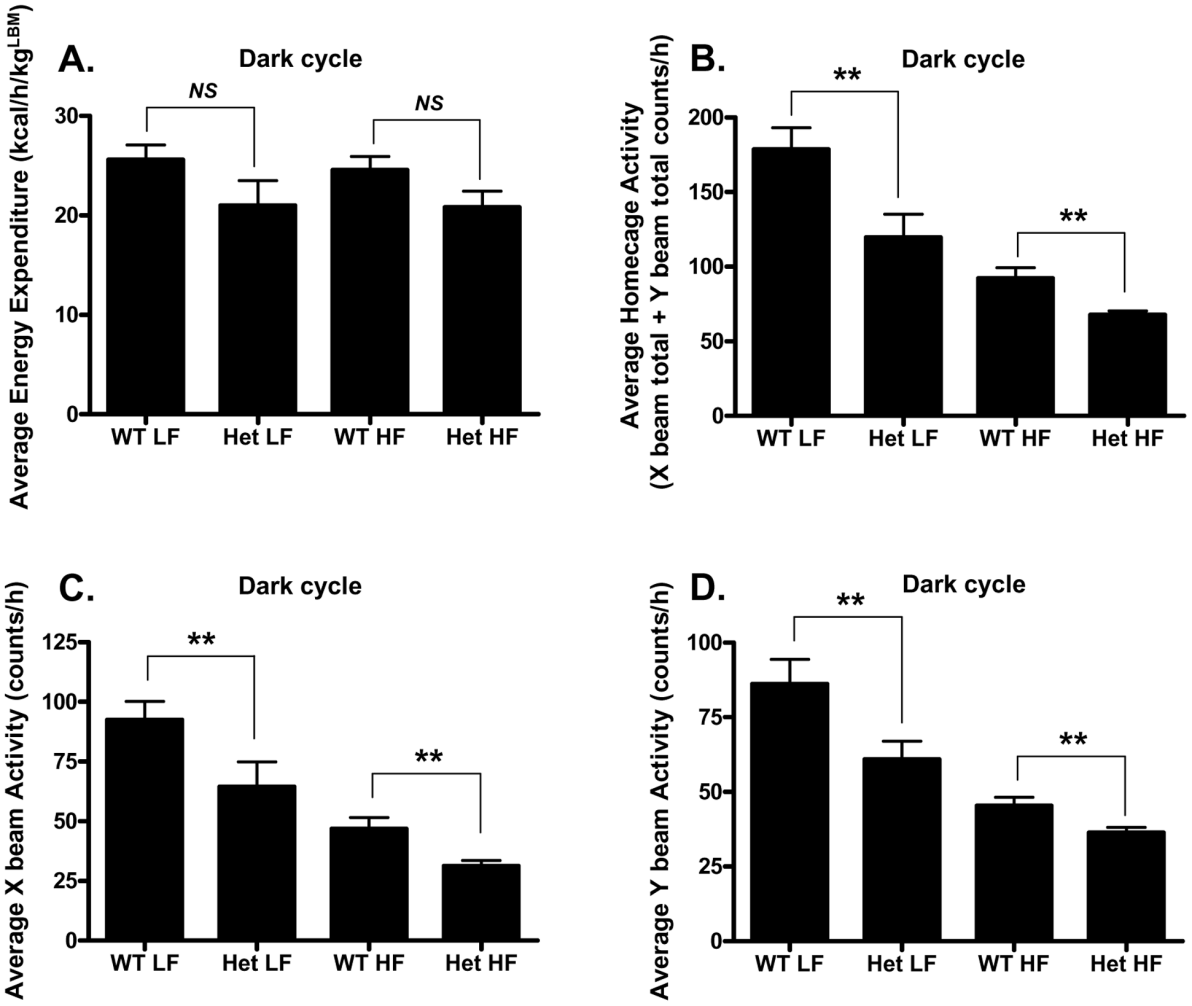
Het GIP Tg mice exhibited decreased physical activity but unaltered energy expenditure. After 11–12 weeks of LF or HF diet feeding, WT and Het GIP Tg mice (*n* = 4∼5/group) were placed in individual cages, and energy expenditure and physical activity were assessed during the dark cycle. **A.** Energy expenditure in LF- and HF diet-fed Het GIP Tg and WT littermates. **B.** Average home cage activity in Het GIP Tg and WT littermates. **C.** Average X beam activity in Het GIP Tg and WT littermates. **D.** Y beam activity in Het GIP Tg and WT littermates. All data represent the mean ± S.E.M. and significance was tested using ANOVA with a Newman-Keuls post hoc test, where ** represents p<0.05 *vs* indicated group; *NS* represents not significant.

In view of the fact that we had previously shown that GIP and insulin act in a synergistic manner to regulate adipocyte function, and to gain a better understanding of the underlying molecular mechanisms involved in the reduced adiposity in GIP Het mice, we determined the relative expression levels of a panel of adipocyte-related genes using quantitative PCR arrays (RT^2^ Profiler PCR Array). As shown in Figures 3G and 3H, a number of genes involved in fatty acid synthesis (ATP citrate lyase, *Acly*), glyceroneogenesis (phosphoenolpyruvate carboxykinase, *Pck1*) and mitochondrial biogenesis and function (PPARγ coactivator 1 α (*Ppargc1a*; *PGC1α*), endothelial nitric oxide synthase 3 (*Nos3*), Uncoupling protein 2 (*Ucp2*)) were down-regulated in Het GIP Tg mice fed the HF diet. Additionally, gene expression of proteins involved in inflammatory signaling pathways were significantly decreased, including angiotensin converting enzyme (*Ace*), glucose-6-phosphate dehydrogenase 2 (*G6pd2*), MCP-1 (*Ccl2*), IKKβ (*Ikbkb*), interleukin 4 receptor alpha (*IL4ra*), TNF receptor superfamily member 1b (*Tnfrsf1b*), and PAI-1 (*serpine1*). Intriguingly, insulin receptor substrate (*Irs-1*) was also decreased in GIP Tg mice (Figure 3G). On the other hand, expression of angiotensinogen (*Agt*), PPARγ (*Pparg*), soluble carrier family 14, member 2 (*Slc14a2*) and *Mapk14* were increased (Figure 3H). Subsequently it was found that adipose tissue protein expression levels of IRS-1 were also decreased in GIP Tg mice, whereas IRS-2 was increased (Figure 3I).

**Figure 6:**
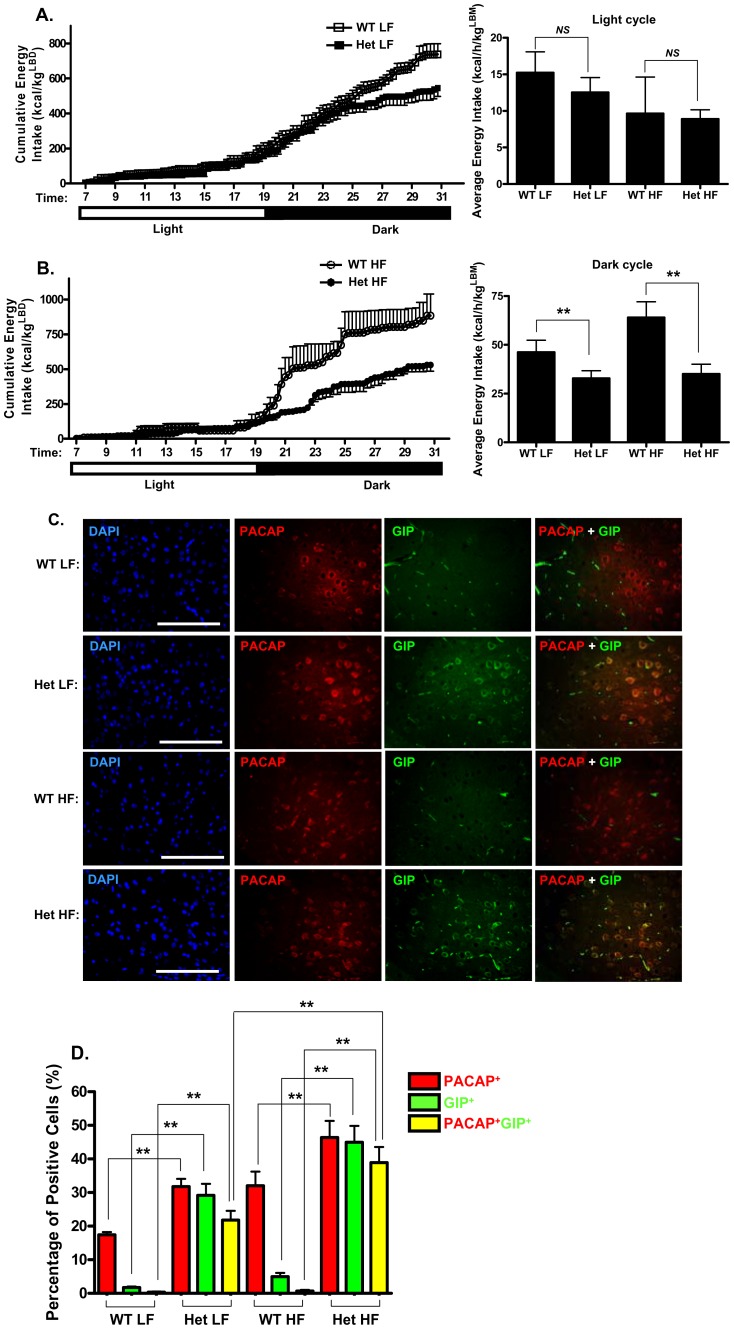
Decreased energy intake in Het GIP Tg mice. After 11–12 weeks of LF or HF diet feeding, WT and Het GIP Tg mice (*n* = 4∼5/group) were placed in individual cages, and energy intake was assessed during the light and dark cycle. **A and B.** Cumulative energy intake in LF (A)- and HF (B) diet-fed Het GIP Tg and WT littermates. **C–D.** Histological analyses of brain tissue from Het GIP Tg and WT littermates following 13 weeks of LF and HF feedings. **C.** Representative images of GIP immunoreactivity in the hypothalamus. Brain sections were stained with GIP (green), PACAP (red) and DAPI (blue). The scale bar indicates 100 µm. **D.** Percentage of positive cells. GIP-, PACAP-, and GIP/PACAP double positive cells were expressed as the percentage of positive cells, respectively. All data represent the mean ± S.E.M. and significance was tested using ANOVA with a Newman-Keuls post hoc test, where ** represents p<0.05 *vs* indicated group; *NS* represents not significant.

### 4. Het GIP Tg Mice were not Resistant to GIP

After 13 weeks of diet feeding, circulating GIP levels in Het GIP Tg mice in LF and HF feeding groups were elevated ∼83.5-fold and ∼41.1-fold over wild type levels, respectively, (Figure 4A). Since the GIP receptor undergoes down-regulation with prolonged exposure to elevated GIP [28], we next determined whether blunted GIP responses contributed to the phenotype demonstrated by Het GIP Tg mice. Exogenous GIP (24 nmol/kg) was therefore administrated to Het GIP Tg mice and their WT littermates with concurrent glucose tolerance tests. As shown in Figures 4B–4E, GIP administration resulted in significantly reduced glycemic excursions relative to the saline control in both Het GIP Tg mice and WT littermates in both LF and HF feeding groups. Therefore, despite the greatly elevated GIP levels, Het GIP Tg mice still responded to GIP indicating that the reduced adiposity, improved insulin sensitivity/glucose tolerance as well as β-cell function in Het GIP Tg mice were not a result of blunted GIP responses.

**Figure 7:**
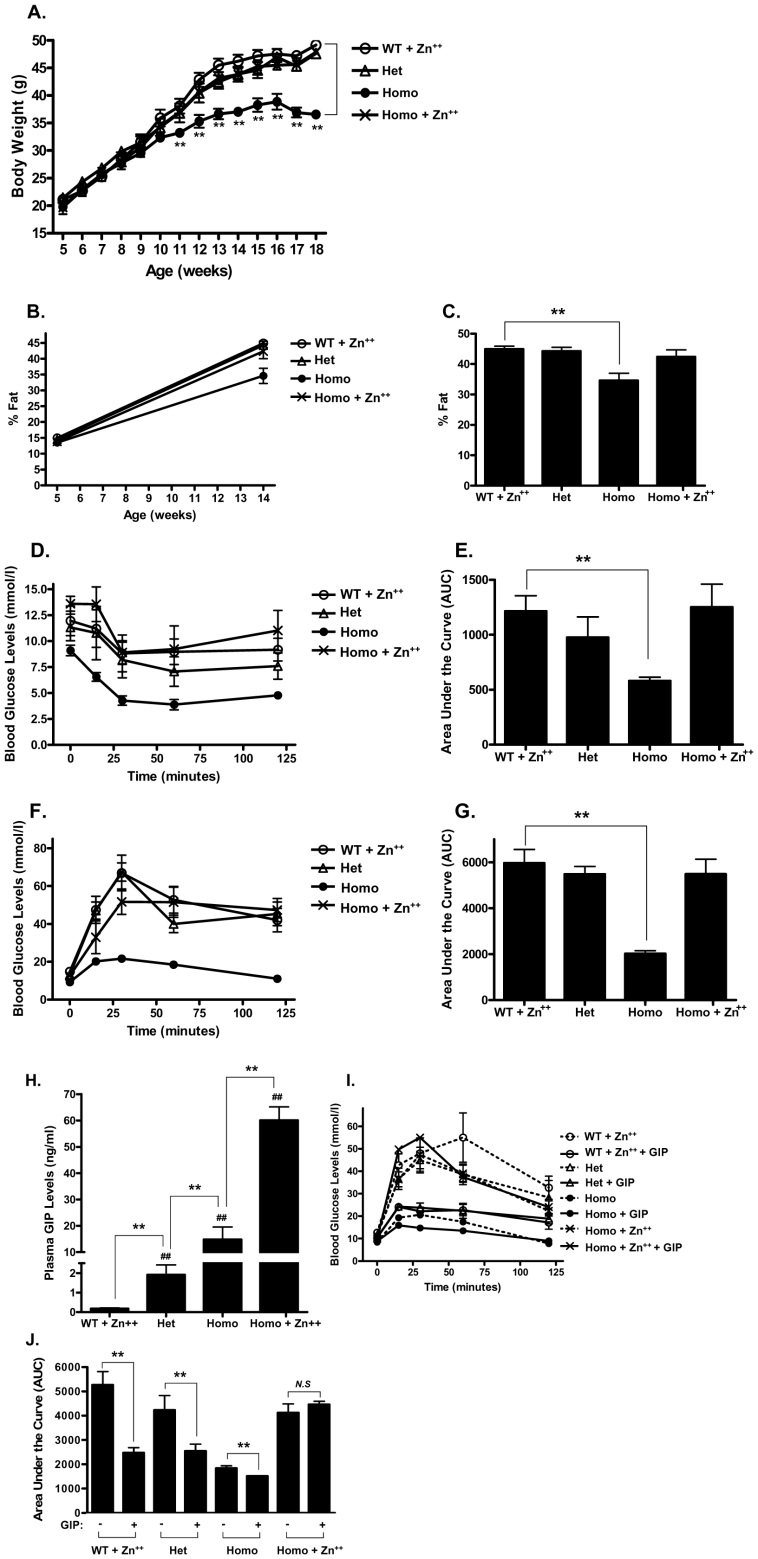
Excessive overexpression ablates GIP effects on HF diet–induced weight gain. A. Body weight gain in Het, Homo GIP Tg and WT mice placed on HF diet in the presence or absence of 25 mM ZnSO_4_. **B.** Percentage fat gain in Het, Homo GIP Tg and WT mice. **C.** Percentage fat of Het, Homo GIP Tg and WT mice on 14 weeks old. **D.** Glucose levels during an insulin tolerance test in HF diet-fed Het, Homo GIP Tg and WT mice in the presence or absence of 25 mM ZnSO_4_. After 7 weeks of HF feeding, Het, Homo GIP Tg and WT mice (12 weeks old, *n* = 4 ∼6/group) were fasted for 4 hours and subsequently injected with 0.75 U/kg insulin. Blood glucose levels were measured at 0, 15, 30, 60 and 120 min following insulin administration. **E.** Quantification of AUC for the total glycemic excursions in **D.** **F.** Glucose levels during a glucose tolerance test in HF diet-fed Het, Homo GIP Tg and WT mice in the presence or absence of 25 mM ZnSO_4_. After 11 weeks of HF feeding, Het, Homo GIP Tg and WT mice (*n* = 4 ∼6/group) were fasted for 4 hours and blood glucose levels measured at 0, 15, 30, 60 and 120 min following the glucose challenge (2 g/kg). **G. **Quantification of AUC for the total glycemic excursions in **F**. **H.** Plasma GIP levels. After 13 weeks of feeding, circulating GIP levels were determined in Het, Homo GIP Tg and WT mice (*n* = 4 ∼6/group). **I.** Effects of GIP on glycemic excursion in HF diet-fed Het, Homo GIP Tg and WT mice. After 13 weeks of feeding, Het, Homo GIP Tg and WT mice (*n* = 4 ∼6/group) were fasted for 4 hours, and PBS or GIP (24 nmol/kg) was administered immediately prior to glucose loading (2 g/kg). Blood glucose levels were measured at 0, 15, 30, 60 and 120 min following the glucose challenge. **J. **Quantification of AUC for the total glycemic excursions in **I**. All data represent the mean ± S.E.M. and significance was tested using ANOVA with a Newman-Keuls post hoc test, where ## represents p<0.05 *vs* WT + Zn^++^, ** represents p<0.05 *vs* indicated group or WT littermates; *NS* represents not significant.

### 5. Indirect Calorimetry Revealed no Significant Differences in Energy Expenditure

Mechanisms involved in the reduced adiposity in GIP Tg mice were next assessed. Since resistance to diet-induced obesity could result from increased energy expenditure and/or decreased food intake, both were examined. Het GIP Tg mice showed a non-significant trend towards decreased energy expenditure (Figure 5A and Figures S1A and S1B) and oxygen consumption (Figures S1C and S1D), during both dark and light phases, compared to WT littermates. As expected, the respiratory exchange rate (RER) decreased to <0.80 with mice on the HF diet, but ratios in Het GIP Tg mice did not differ from WT littermates (Figures S1E and S1F). In agreement with this conclusion, Het GIP Tg mice in both LF and HF feeding groups showed significantly decreased, rather than increased, total home cage activity, including both X and Y beam activities at dark cycle, compared to WT littermates (Figures 5B–5D). Apart from Y beam activity, physical activities of Het GIP Tg mice at light cycle did not differ from WT control mice (Figures S2A–S2F).

### 6. Het GIP Tg Mice Exhibited Reduced Energy Intake

Het GIP Tg mice showed significant reductions in food intake during most weeks over the course of the study (Figure S3), and as shown in Figures 6A and 6B, metabolic cage studies showed that these mice demonstrated a marked reduction in energy intake (food intake in kcal) during the dark phase compared to WT littermates on both LF and HF diets. On HF diet, the intake of Tg mice reached ∼54.8% of that of the WT mice following 11–12 weeks of HF feeding and the overall reduction in energy balance would likely account for the decrease in body weight.

Since GIP mRNA was previously reported to be overexpressed in the brain of GIP Tg mice [29], and the hypothalamus is critical for the regulation of food intake-controlling neural circuits, we considered it possible that transgenic GIP expression in the brain could be involved in the modulation of energy intake in GIP Tg mice. Immunohistochemical analyses demonstrate that GIP expression was greatly increased in the ventromedial nucleus (VMN) of the hypothalamus of Het GIP Tg mice on both LF and HF diets, compared with WT littermates (Figures 6C and 6D), mainly co-expressed in pituitary adenylate cyclase-activating polypeptide (PACAP)-positive neurons. The number of GIP/PACAP double positive neurons were significantly increased in Het GIP Tg mice on HF, compared to those on a LF diet (Figures 6C and 6D). Together, these results suggest that transgenic GIP overexpression in the hypothalamus contributed significantly to the reduced energy intake in GIP Tg mice.

### 7. GIP Effects on HF Diet–induced Insulin Resistance and Glucose Intolerance were Plasma Concentration-dependent

To evaluate the effect of varying circulating GIP concentrations on metabolic phenotype, homozygous (Homo, Tg/Tg) GIP Tg mice were screened by progeny testing. One group of Homo GIP Tg mice and WT littermates placed on HF diet in the presence of 25 mM ZnSO_4_ (Zn^2+^) in the drinking water for 13 weeks beginning at 5 weeks of age. The second group of Homo and Het GIP Tg mice fed the same HF diet in the absence of Zn^2^. This resulted in GIP levels at 18 weeks of only 1.9±0.5 ng/ml in the Het GIP Tg mice in the absence of Zn^2+^ (Figure 7H), as compared to 14.8±5.1 ng/ml in the presence of Zn^2+^ (Figure 4A). GIP levels in Homo and Homo + Zn^2+^ were 16.3±4.8 ng/ml and 60.1±5.1 ng/ml, respectively (Figure 7H). In agreement with an earlier study [29], Het and Homo GIP Tg mice demonstrated significantly enhanced GIP levels even in the absence of Zn^2^, due to the “leaky” metallothionein promoter, compared to the WT littermates (Figure 7H). As shown in Figure 7A, Homo GIP Tg mice without Zn^2+^ demonstrated markedly reduced body weight at 13 weeks, compared to the other three groups, due to reduced adiposity (Figures 7B and 7C), but no difference in lean mass (Data not shown). Subsequent studies demonstrated that Homo GIP Tg mice without Zn^2+^ showed significantly improved insulin sensitivity (Figures 7D and 7E) and glucose tolerance (Figures 7F and 7G) compared to WT littermates. The Homo GIP Tg mice with Zn^2+^ were GIP-resistant (Figures 7I and 7J). Together, these results suggest that chronically raising levels of GIP to ∼15 ng/ml are necessary to achieve beneficial effects on glucose homeostasis and adiposity, but more elevated levels result in GIP resistance.

## Discussion

Obesity and associated increases in circulating free fatty acids (FFA) have long been considered major contributors to insulin resistance and glucose intolerance. However, ectopic deposition of triglyceride (TG) is now recognized as an important contributor and adipose tissue expansion has been suggested to be an adaptive response to increased food intake, since it protects against accumulation of excess fat in sites such as the liver and muscle [30]. In a recent study, it was demonstrated that overexpression of adiponectin in mice on an *ob/ob* genetic background resulted in greater obesity, compared to non-transgenic animals, but with marked improvements in both glucose tolerance and insulin resistance, as well as reduced circulating TG and hepatic steatosis [31]. Storage of fat preferentially in adipose tissue and partitioning it away from other insulin-sensitive tissues is therefore probably a major determinant of insulin sensitivity and normal glucose tolerance, an effect of thiazolidinediones that has been termed: ‘lipid steal’ [32]. There may be additional advantages in adipose tissue expansion, including the production of beneficial adipokines such as adiponectin, and the anabolic effects of GIP on the adipocyte may also be physiologically beneficial.

In view of the glucose intolerance but resistance to diet-induced obesity observed in GIPR knockout mice [22], [33]–[34], the current study was initiated to test the hypothesis that chronically elevating GIP levels *in vivo* would increase adipose tissue expansion and exert beneficial effects on glucose homeostasis. In line with earlier studies demonstrating that subcutaneous administration of long-acting GIP analogues resulted in improved β-cell function and glycemic control [10], [11], [13], GIP Tg mice showed improved insulin sensitivity, glucose tolerance and β-cell function (Figures 1 and 2), as well as reduced steatosis. Despite greatly elevated circulating GIP levels, the improved metabolic profile was not associated with blunted GIP responsiveness (Figure 4). However, greatly elevated levels of GIP in homozygous Tg mice treated with ZnSO_4_ did induce resistance (Figures 7I and 7J), suggesting that there is a circulating concentration threshold, above which the beneficial effects of GIP on metabolism are lost.

In contrast to our initial hypothesis regarding adipose tissue expansion, chronic overexpression of GIP in mice reduced the development of adiposity in response to a HF diet (Figure 1B). Importantly, eWAT from Het GIP Tg mice exhibited substantially decreased macrophage infiltration (Figures 3E and 3F). In order to examine whether this was associated with altered adipose tissue gene expression profiles, eWAT was examined and a number of genes encoding proteins involved in mitochondrial function and inflammatory signaling pathways were found to be down-regulated in Het GIP Tg mice on a HF diet (Figure 3G). This included reductions in expression of the proinflammatory cytokine MCP-1 (*Ccl2*), plasminogen activator inhibitor type 1 (*Serpin1*) and interleukin 4 receptor alpha (*IL4ra*), as well as a component of the NFκB pathway, Iκb kinase β (*Ikbkb*). Altered expression of enzymes associated with ROS production may also be linked to reduced inflammation. For example, decreased G6pd2 would be expected to deprive the cell of NADPH that is needed for both ROS and NO production [35]. Also of interest was the reduced expression of the angiotensin converting enzyme gene (*Ace*) since angiotensin II is involved in increasing fat deposition [36], activation of nitric oxide synthase 3 (eNos) and modulating the macrophage phenotype [37]. The increase in angiotensinogen gene (*Agt*) expression may have been a compensatory response to the decrease in Ace activity. The reduction in *Nos3* expression and consequent lowering of NO production could be related to anti-inflammatory effects of GIP and/or altered mitochondrial biogenesis and function. eNos has been linked to the induction of PGC-1α expression [38]–[39] and both PGC-1α [40] and Ucp2 [41] are involved in mitochondrial biogenesis, reduction of which may contribute to lowering ROS production. In contrast, the p38 MAPKα gene (*Mapk 14*) was up-regulated and mitochondrial biogenesis is reduced by p38 MAPK activation [42], a target of GIP signaling. The marked reduction in expression of genes coding for proinflammatory proteins and enzymes involved in mitochondrial biogenesis was unexpected, since previous studies showed that GIP stimulated adipocyte resistin release [16], an effect that has been interpreted as pro-inflammatory. However, it is possible that resistin responses to GIP are unrelated to inflammatory effects, but associated with resistin’s metabolic action on the adipocyte.

Expression levels of adipocyte enzyme genes involved in fat metabolism were also decreased in Het GIP Tg mice. Down-regulation of *Ppargc1a* and *Acly* would be expected to reduce fatty acid synthesis, whereas reduced *Pck1* expression would lower rates of glyceroneogenesis and reesterification [43], [44]. In contrast *Pparg* expression was increased. Apart from *Pparg* these changes were also unexpected because *in vitro* treatment of 3T3-L1 adipocytes and human or rodent adipocytes with GIP has been shown to induce lipogenesis [15]–[17], [21]. However, lipogenic responses to GIP are insulin-dependent and, since IRS-1 was down-regulated and IRS-2 increased, the synergistic effects of these hormones may be altered.

It is therefore likely that reduced steatosis and adipocyte macrophage infiltration plus altered WAT signaling all contributed to the improved metabolic profiles of the Het GIP Tg mice on the HF diet, including the improved insulin sensitivity. We next examined whether altered physical activity, energy expenditure or food intake contributed to the weight loss. Het GIP Tg mice showed significantly decreased physical activity, including homecage activity and X and Y beam activities during the dark cycle, when compared to WT littermates on both LF and HF diets (Figures 5B, 5C and 5D). As would be anticipated, these changes in locomotor activity were opposite to those reported for GIPRKO mice [22], [34]. No significant differences in energy expenditure, determined by indirect calorimetry, were apparent between Het GIP Tg mice and WT littermates on either LF or HF diets (Figure 5A and Figure S1), whereas higher energy expenditure was reported for GIPRKO mice [22]. This apparent discrepancy may be partly due to the fact that energy expenditure was normalized to lean body mass (LBM), as recently recommended [45], since a unit of fat mass has a lower level of oxygen consumption than a unit of lean mass [45]. Overall these results indicated that changes in homecage activity and energy expenditure did not contribute to the reduced weight gain of Het GIP Tg mice and that the metabolic phenotype resulted from increased GIP expression and not blunted GIP responsiveness. Het GIP Tg mice did, however, exhibit a marked reduction in energy intake compared to WT littermates on both LF and HF diets, that was delayed in the light phase but apparent from the onset of the dark phase (Figures 6A and 6B). Reduced adiposity in Het GIP Tg mice was therefore at least partially due to reduced energy intake.

The hypothalamus integrates and orchestrates neural, metabolic and humoral signals from the periphery and multiple hypothalamic neural pathways are involved in the control of energy intake. A number of effects of GIP on central nervous functions have been demonstrated, including improved cognition, hippocampal synaptic plasticity and glucose homeostasis [46], whereas GIPRKO mice demonstrated impairments in learning, synaptic plasticity and neurogenesis [47]. GIP was reported to be highly expressed in the hippocampus and thalamus of adult rat brain, while its expression in rat hypothalamus was relatively low [48], and it was previously shown that GIP expression in the brain was increased in the GIP Tg mouse strain [29], [49]. The VMN is a major site for PACAP biosynthesis [50], with mRNA expression regulated by energy status [51]. In the current study, GIP was found to be strongly co-expressed in this region in Het Tg mice on both LF and HF diets, with significantly increased numbers of GIP/PACAP double positive neurons on HF feeding (Figures 6C and 6D). Although VMN application of GIP does not appear to have been performed to date, there is therefore a high possibility that changes in central expression of GIP contributed to the reduced food intake in the GIP overexpressing mice.

One of the remaining questions arising from this study is the relative contribution of central and peripheral GIP to the phenotype of the overexpressing mice. Subcutaneous administration of D-Ala^2^-GIP_1–30_ to Zucker diabetic fatty rats resulted in significantly improved glucose tolerance and insulin sensitivity, with a mild effect on food intake [13]. However, in carefully controlled studies, subcutaneous administration of GIP1-42 or GIP1-30 in HF fed mice was not found to have any major effects on food intake or body weight, despite significantly improved metabolic profiles [11]. Additionally, administration of a range of long acting GIP analogues, including N-acGIP, GIP (Lys^37^MYR), N-AcGIP(Lys^37^MYR) and N-AcGIP(LysPAL^37^), had no effect on body weight and food intake when compared to saline-treated controls [52]–[53]. This suggests that reduced food intake in GIP Tg mice resulted mainly in response to the hypothalamic expression and action of GIP. However, peripherally administrated GIP analogues have recently been reported to cross the blood-brain-barrier and exert central effects in mice [46], [54]. It is therefore possible that the high circulating levels of GIP in the transgenic mice and the duration of GIP overexpression contributed to the reduced food intake. The almost complete ablation of steatosis was probably secondary to the reduction in adiposity. However, in view of the more robust changes in the pancreatic β-cells and adipose tissue (∼68.5% decrease of TUNEL+ β-cells, ∼58.6% decrease of crown-like structure content *vs* ∼6.1% decrease in fat mass in Het GIP Tg mice), the improved β-cell function, altered WAT gene expression pattern as well as the decreased macrophage infiltration likely involved direct effects of GIP on these tissues. Further expanded studies will be required to clearly delineate central *vs* peripheral effects of GIP, and their potential direct/indirect contribution to GIPR agonist action.

In summary, we have shown that GIP Tg mice exhibit enhanced β-cell function, resulting in improved glucose tolerance and insulin sensitivity. Lower body weight was linked to reduced energy intake, that probably resulted mainly from overexpression of hypothalamic GIP in the Tg mice. They were also characterized by altered expression of adipocyte genes involved in the regulation of mitochondrial function and inflammation. Together, our findings support chronically elevating transgenic GIP expression exerts beneficial effects on a number of metabolic factors that are linked to the pathology of obesity and type 2 diabetes, and suggest that potential hypothalamic effects of transgenic GIP on food intake are worthy of further study.

## Experimental Procedures

### Animals and Genotyping

Male GIP-overexpressing transgenic mice developed at the Medical College of Georgia, Augusta, GA [29] were crossed with female C57BL/6, and rederived in the Centre for Disease Modeling (CDM), University of British Columbia. GIP-overexpressing transgenic mice were generated using a gene construct containing the GIP coding sequence flanked by regulatory regions from the endogenous metallothionein locus (MTI and MTII) [55], resulting in a construct that retains proper regulation with limited artifactual effects derived from the site of integration. The metallothionein promoter drives ubiquitous gene expression but levels of transgene expression differ depending on the target tissue [55]. Genotyping was performed at weaning with genomic DNA obtained from ear notched biopsies. The duplex PCR primer sequences used for the genotyping were as follows: forward primer for GIP, 5′-TCT GTT GCT GGT GCT CCT GTT-3′; reverse primer for GIP, 5′-ATC ACT GAG GCT CTT GGG CAA-3′; forward primer for INS-2, 5′-AGT GGC ACA ACT GGA GCT GG-3′; reverse primer for INS-2, 5′-TAT TCA TTG CAG AGG GGT AGG C-3′. Heterozygous offspring of the founders (Het, Tg/+) was further crossed with nontransgenic wild-type (WT, +/+) littermates. At 5 weeks of age, resulting Het GIP transgenic (GIP Tg) mice and non-transgenic WT littermates offspring were placed on either a low fat (LF) diet (D12450B, Research Diets Inc., New Brunswick, NJ) composed of 10, 70 and 20 kcal% of fat, carbohydrate and protein or high fat (HF) diet (D12492, Research Diets Inc) composed of 60, 20 and 20 kcal% of fat, carbohydrate and protein. For induction of GIP expression, 25 mM ZnSO_4_ was added to the drinking water of Het and non-transgenic WT littermates. For the second part of the experiment, (Figures 7), Het mice were further crossed with Hets to generate homozygous transgenic mice (Homo, Tg/Tg). Progeny testing was performed and homozygosity confirmed by outcrossing presumptive Homo mice to nontransgenic WT littermates and checking progeny for 100% transmission of the transgene. Circulating plasma GIP levels were determined using a Rat/Mouse total GIP ELISA kit (Millipore). Resulting Het, Homo GIP Tg mice and non-transgenic WT littermates (5 weeks old) were placed on HF diet in the presence or absence of 25 mM ZnSO_4_ in the drinking water, as indicated in the Figure legends. All animal experiments were specifically approved by the University of British Columbia Committee on Animal Care and Canadian Council on Animal Care.

### DEXA (Dual Energy X-ray Absorptiometry) Scanning

Tissue composition of the mice was determined at 5 and 14 weeks, using a Lunar PIXImus2 densitometer (Outside Sales, Madison WI). Shortly before scanning, mice were anesthetized by isoflurane inhalation, placed on the plastic tray of the scanner bed in a prone position and a nose cone was fitted over the face to maintain isoflurane anesthesia throughout the procedure. DEXA scans were analyzed based on manufacturer’s algorithms, using the PIXImus2 software. Values for total tissue mass (g), fat content (g), lean content (total tissue mass minus fat content, g) and percentage fat (fat content divided by total tissue mass) were obtained.

### Blood Glucose Determinations, Intraperitoneal Glucose Tolerance Tests (IPGTTs), Intraperitoneal Insulin Tolerance Tests (IPITT), GIP-Glucose Tolerance Tests (GIP-GTT), and Plasma Hormone Measurements

Fasting and non-fasting blood glucose levels were measured in blood collected from the saphenous vein using a Freestyle Glucometer (Abbott Laboratories) at the time points indicated in Figures 1, 2, 4 and 7. For the IPGTTs and IPITTs, mice were fasted for 4 hr and blood glucose levels measured at 0, 15, 30, 60 and 120 min following the glucose (2 g/kg) or insulin challenge (0.75 U/kg). For the GIP-GTT, mice were fasted for 4 hr and PBS or GIP (24 nmol/kg) were administered immediately prior to glucose loading (2 g/kg). Blood glucose levels were measured as above. Blood samples with glucose levels ≥27.8 mmol/l were diluted with control solution provided by the manufacturer (Abbott Laboratories) and levels calculated. Plasma insulin, glucagon and GIP levels were determined using a multi-spot® assay kit (Meso Scale Discovery, Gaithersburg, MD) and Rat/Mouse total GIP ELISA kit (Millipore), respectively.

### Morphological Analyses of Liver and Epididymal White Adipose Tissue

Livers and epidydimal fat pads were harvested and fixed in 10% neutral buffered formalin. Sections obtained from paraffin-embedded tissues were stained with Hematoxylin & Eosin (H & E) using standard protocols. Fat sections were incubated with antibody against the mouse macrophage antigen MAC-2 and visualized with FITC conjugated anti-rat secondary antibody. Cell nuclei were counterstained with DAPI (4′, 6- diamino-2-phenylindole) and imaged using a Zeiss laser scanning confocal microscope (Axioskop2). MAC-2 positive crown-like structures (CLS) per field were counted manually and the number of CLS per 1000 nuclei was used as a measure of adipose tissue CLS content. Liver sections were incubated with the lipid body stain BODIPY 493/503 and counterstained with DAPI. Intensity of signals was quantified by computer-based densitometric analysis using the Northern Eclipse program (ver.6).

### RT^2^ Profiler™ PCR Arrays

Total RNA was extracted from epididymal fat tissues using an RNeasy Lipid Tissue Mini Kit (Qiagen), and cDNA fragments were generated by reverse transcription using RT^2^ First Strand Kit (SAbiosciences). RT^2^ Profiler PCR Arrays were performed to simultaneously quantify mRNAs of 84 genes associated with insulin signaling plus housekeeping genes, according to the manufacturer’s protocol. Average expression of the five housekeeping genes on the array was used to normalize the gene expression by the ΔΔCt method.

### Western Blot Analysis

Total cellular extracts from each sample were separated on a 13% sodium dodecyl sulfate (SDS)/polyacrylamide gel and transferred onto nitrocellulose membranes (Bio-Rad Laboratories, Mississauga, ON). Probing of the membranes was performed with IRS-1, IRS-2 and β-actin antibodies. Immunoreactive bands were visualized by enhanced chemiluminescence (Millipore Millipore, Billerica, MA) using horseradish peroxidase-conjugated IgG secondary antibodies.

### Immunohistochemistry

Pancreatic sections from GIP Tg and WT littermates were subjected to double immunostaining for insulin and glucagon, with apoptosis staining in adjacent sections. Following deparaffinization, the sections were incubated with insulin and glucagon antibodies and visualized with Texas Red® dye conjugated anti-rabbit secondary antibody and Alexa fluor® 488 conjugated anti-mouse antibody. Cell nuclei were counterstained with DAPI (4′, 6-diamino-2-phenylindole) and imaged using a Zeiss laser scanning confocal microscope (Axioskop2). TUNEL assay was performed in adjacent sections to detect DNA fragmentation in apoptotic cells using the Apoptag® apoptosis detection kit (Intergen Company, Purchase, NY), according to the manufacturer’s instructions. β-cell area was measured on the sections of each pancreas stained for insulin. Morphometric evaluation of the total β-cell area was performed by computer-assisted image analysis (Northern Eclipse ver. 6 and NIH Image J) using light and immunofluorescence microscopy. The area of insulin-positive cells and the total area of the tissue section were evaluated for each section, and the percentage β-cell area was calculated by the ratio of the area occupied by insulin-positive cells to the total pancreatic area.

### Measurement of Total Pancreatic Insulin Content

Pancreata were homogenized in 5 ml of ice-cold 2 N acetic acid, boiled and centrifuged (10 min, 15,000 rpm, 4°C). The supernatant was then assayed for insulin content and normalized for protein concentration (BCA; Pierce, Rockford, IL).

### Indirect Calorimetry

Mice were individually housed, with free access to food and water, and acclimated in metabolic chambers. The following parameters were continuously recorded with measurements taken every 15 minutes: Gas exchange (VO2 and VCO2), homecage activity, water intake and food intake. Physical activity was measured by utilizing infrared beams to monitor an animal’s homecage movement in the X, Y, and Z axis. Following metabolic chamber recordings, DEXA scanning was performed to determine lean body mass (LBM). VO2, VCO2 and energy expenditure were calculated according to the manufacturer’s guidelines (PhenoMaster Software, TSE Systems, Bad Homburg, Germany) and normalized to LBM. The respiratory exchange ratio (RER) was estimated by calculating the ratio of VCO2/VO2.

### Statistical Analysis

Data are expressed as means ± Standard Errors of the Mean (SEM). Area under the curves (AUCs) were calculated using the algorithm provided in the PRISM software package (GraphPad, San Diego, CA). Significance was tested using analysis of variance (ANOVA) with Newman-Keuls hoc test (*P<*0.05) or Student’s *t* test (*P<*0.05) as indicated in figure legends.

## Supporting Information

Figure S1 **Het GIP Tg mice didn’t exhibit increased energy expenditure.** After 11–12 weeks of feeding, WT and Het GIP Tg mice (16–17 weeks old, *n* = 4∼5/group) were placed in individual cages, and physical activity was assessed during the light and dark cycle. **A and B.** Energy expenditure in LF (**A**)- and HF (**B**) diet-fed Het GIP Tg and WT littermates. **C and D.** Oxygen consumption in LF (**C**)- and HF (**D**) diet-fed Het GIP Tg and WT littermates. **E and F.** Respiratory exchange rate in LF (**E**)- and HF (**F**) diet-fed Het GIP Tg and WT littermates. All data represent the mean ± S.E.M. and significance was tested using ANOVA with a Newman-Keuls post hoc test, where ** represents p<0.05 *vs* indicated group; *N.S.* represents not significant.
(TIF)

Figure S2 **Decreased homecage activity in Het GIP Tg mice.** After 11–12 weeks of feeding, WT and Het GIP Tg mice (16–17 weeks old, *n* = 4∼5/group) were placed in individual cages, and physical activity was assessed during the light and dark cycle. **A and B.** Home cage activity in LF (**A**)- and HF (**B**) diet-fed Het GIP Tg and WT littermates. **C and D.** X beam activity in LF (**C**)- and HF (**D**) diet-fed Het GIP Tg and WT littermates. **E and F.** Y beam activity in LF (**E**)- and HF (**F**) diet-fed Het GIP Tg and WT littermates. All data represent the mean ± S.E.M. and significance was tested using ANOVA with a Newman-Keuls post hoc test, where ** represents p<0.05 *vs* indicated group; *N.S.* represents not significant.
(TIF)

Figure S3 **Decreased food intake in Het GIP Tg mice.** Het GIP Tg and WT littermates were placed on high fat (HF) diet, 25 mM ZnSO_4_ was added to the drinking water of both Het GIP Tg and WT mice, and food intake was assessed. All data represent the mean ± S.E.M. and significance was tested using Student’s *t* test, where ** represents p<0.05 *vs* WT HF.
(TIF)
