# Supplementary figures and images for: GIP-Overexpressing Mice Demonstrate Reduced Diet-Induced Obesity and Steatosis, and Improved Glucose Homeostasis
Source: PLoS One. 2012 Jul 3;7(7):e40156. doi: 10.1371/journal.pone.0040156 (PMC3388996; doi:10.1371/journal.pone.0040156)

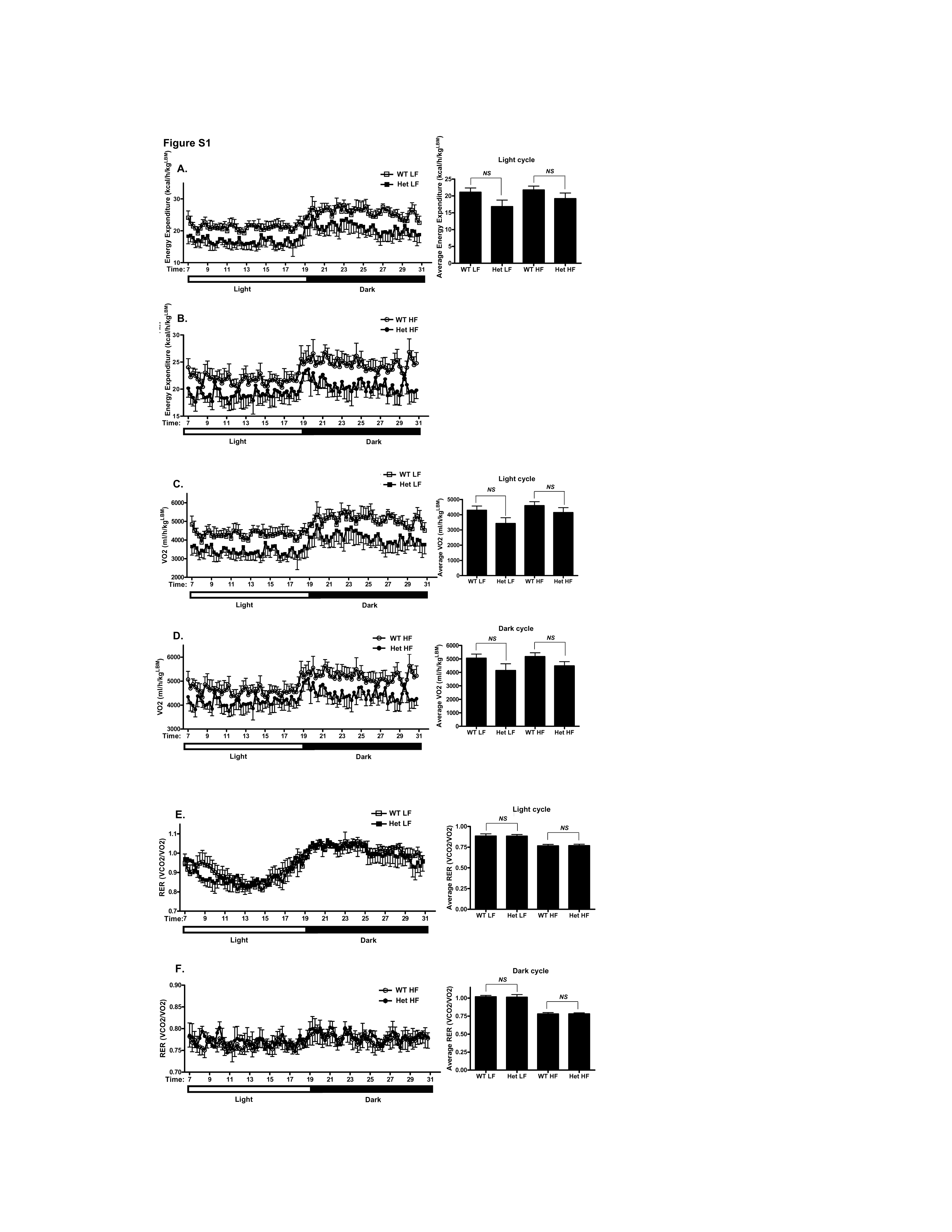

Supplement: Figure S1 — Het GIP Tg mice didn’t exhibit increased energy expenditure. After 11–12 weeks of feeding, WT and Het GIP Tg mice (16–17 weeks old, n = 4∼5/group) were placed in individual cages, and physical activity was assessed during the light and dark cycle. A and B. Energy expenditure in LF (A)- and HF (B) diet-fed Het GIP Tg and WT littermates. C and D. Oxygen consumption in LF (C)- and HF (D) diet-fed Het GIP Tg and WT littermates. E and F. Respiratory exchange rate in LF (E)- and HF (F) diet-fed Het GIP Tg and WT littermates. All data represent the mean ± S.E.M. and significance was tested using ANOVA with a Newman-Keuls post hoc test, where ** represents p<0.05 vs indicated group; N.S. represents not significant. (TIF) [file pone.0040156.s001.tif]

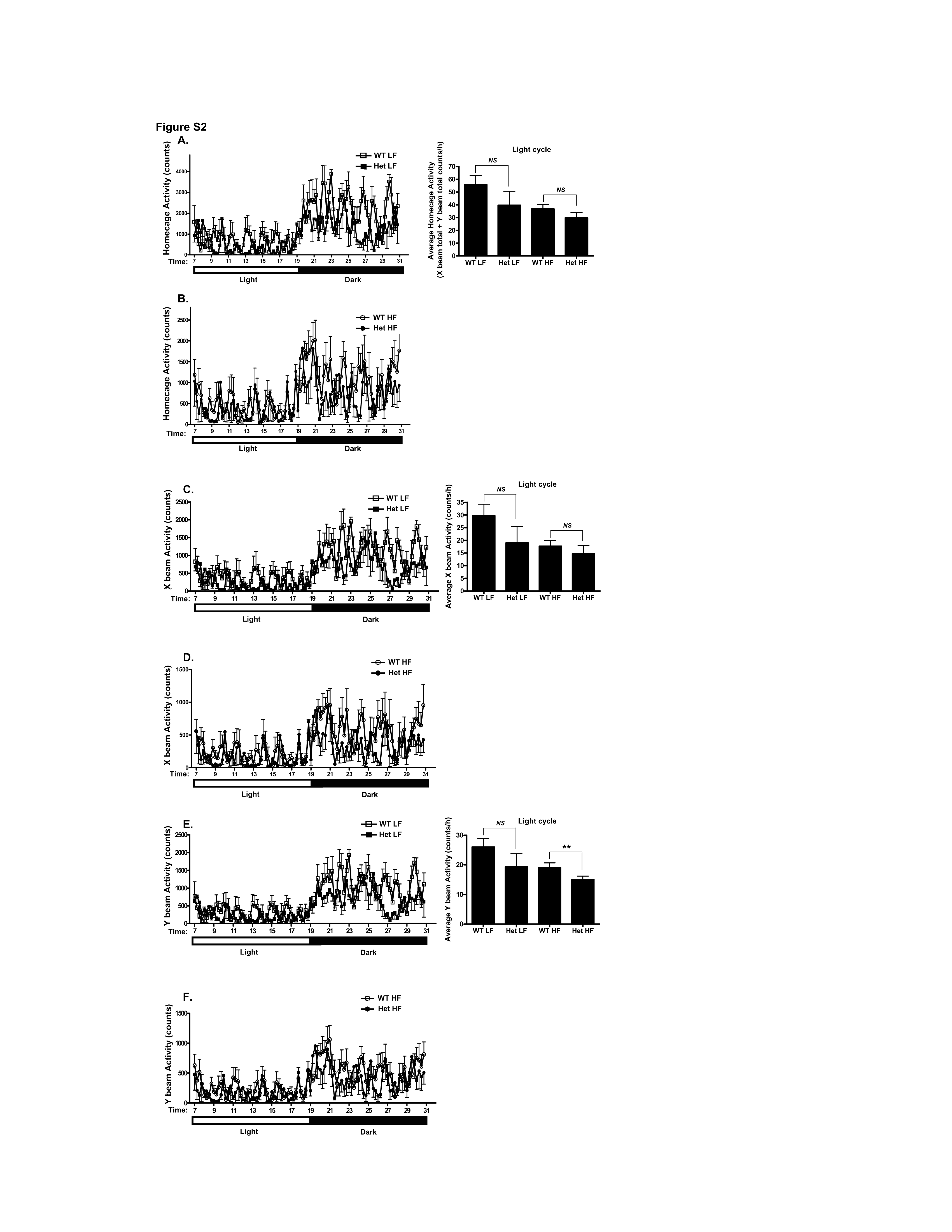

Supplement: Figure S2 — Decreased homecage activity in Het GIP Tg mice. After 11–12 weeks of feeding, WT and Het GIP Tg mice (16–17 weeks old, n = 4∼5/group) were placed in individual cages, and physical activity was assessed during the light and dark cycle. A and B. Home cage activity in LF (A)- and HF (B) diet-fed Het GIP Tg and WT littermates. C and D. X beam activity in LF (C)- and HF (D) diet-fed Het GIP Tg and WT littermates. E and F. Y beam activity in LF (E)- and HF (F) diet-fed Het GIP Tg and WT littermates. All data represent the mean ± S.E.M. and significance was tested using ANOVA with a Newman-Keuls post hoc test, where ** represents p<0.05 vs indicated group; N.S. represents not significant. (TIF) [file pone.0040156.s002.tif]

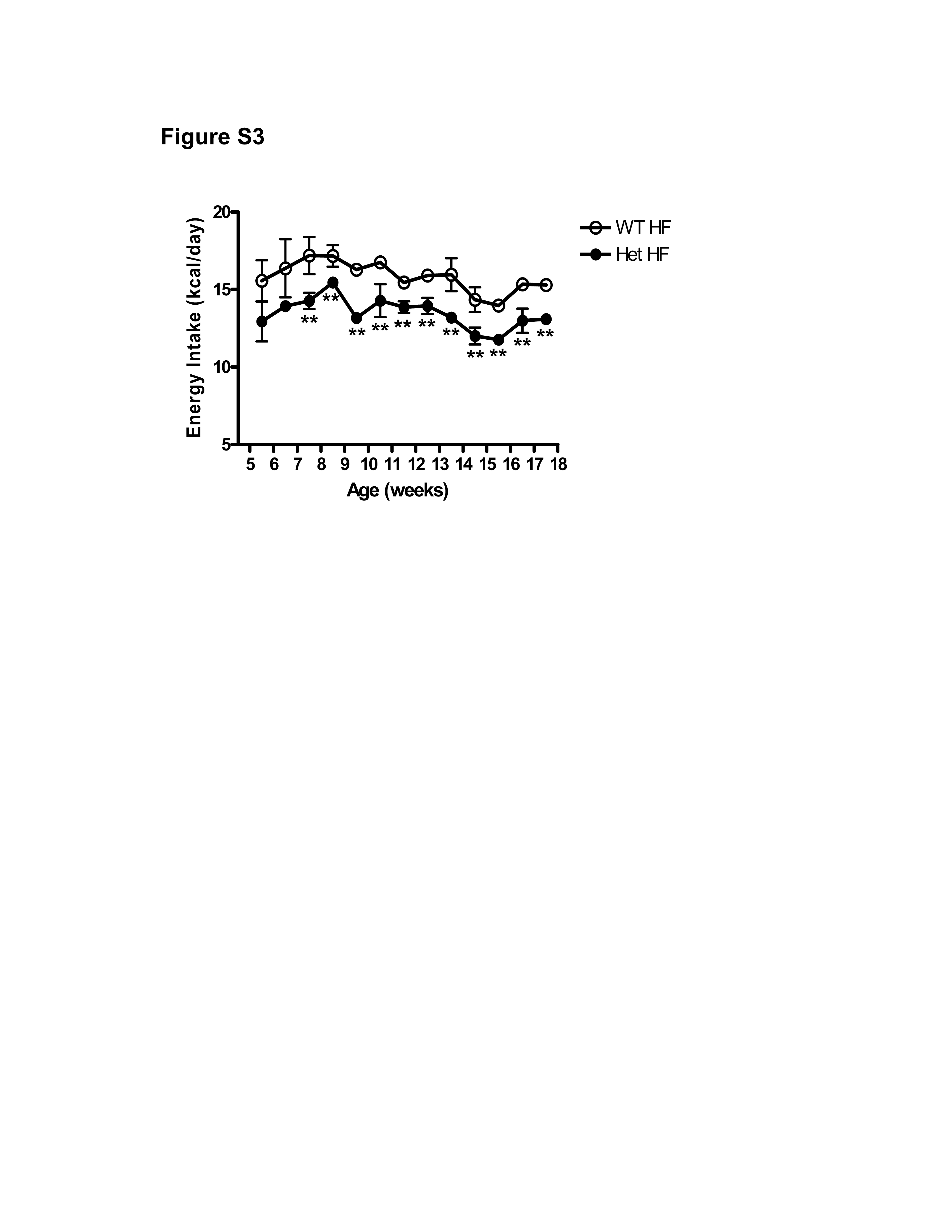

Supplement: Figure S3 — Decreased food intake in Het GIP Tg mice. Het GIP Tg and WT littermates were placed on high fat (HF) diet, 25 mM ZnSO4 was added to the drinking water of both Het GIP Tg and WT mice, and food intake was assessed. All data represent the mean ± S.E.M. and significance was tested using Student’s t test, where ** represents p<0.05 vs WT HF. (TIF) [file pone.0040156.s003.tif]
